# Cryo-EM Structure of the *Rhodobacter sphaeroides* Light-Harvesting 2 Complex at 2.1 Å

**DOI:** 10.1021/acs.biochem.1c00576

**Published:** 2021-10-26

**Authors:** Pu Qian, David J. K. Swainsbury, Tristan I. Croll, Pablo Castro-Hartmann, Giorgio Divitini, Kasim Sader, C. Neil Hunter

**Affiliations:** † Materials and Structural Analysis, Thermo Fisher Scientific, Achtseweg Noord 5, 5651 GG Eindhoven, Netherlands; ‡ Cambridge Institute for Medical Research, University of Cambridge, Cambridge CB2 0XY, U.K.; § Department of Materials Science and Metallurgy, University of Cambridge, Cambridge CB3 0FS, U.K.; ∥ Department of Molecular Biology and Biotechnology, University of Sheffield, Sheffield S10 2TN, U.K.

## Abstract

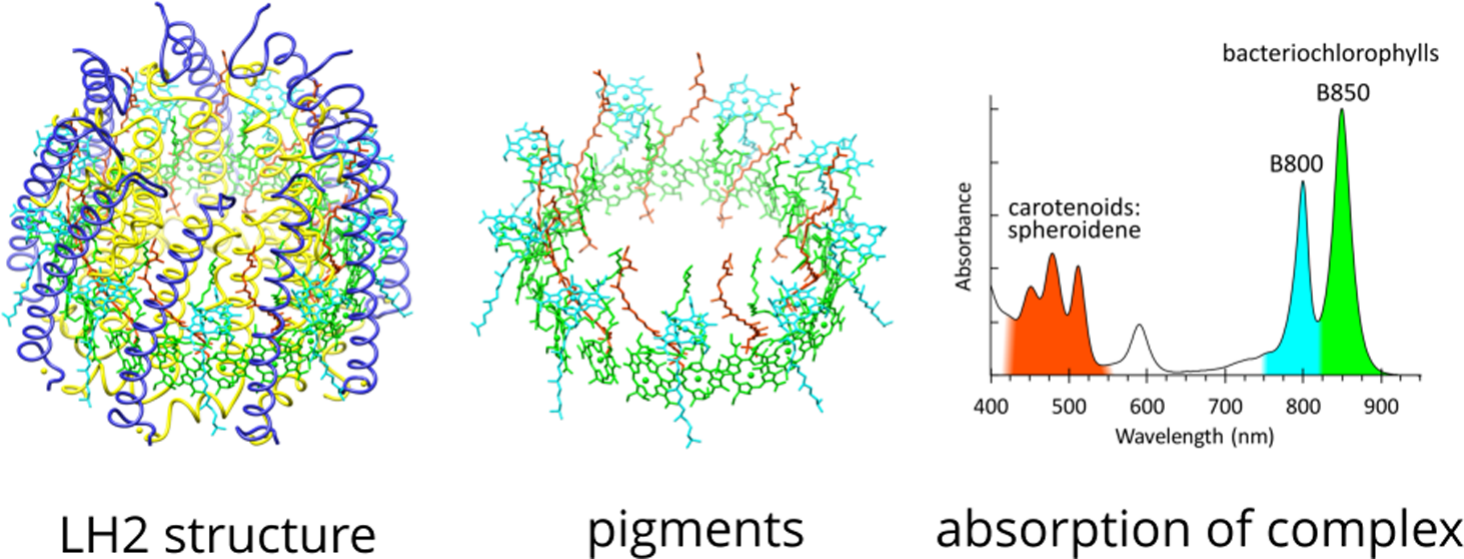

Light-harvesting 2 (LH2) antenna complexes augment the collection of solar energy in many phototrophic bacteria. Despite its frequent role as a model for such complexes, there has been no three-dimensional (3D) structure available for the LH2 from the purple phototroph *Rhodobacter sphaeroides*. We used cryo-electron microscopy (cryo-EM) to determine the 2.1 Å resolution structure of this LH2 antenna, which is a cylindrical assembly of nine αβ heterodimer subunits, each of which binds three bacteriochlorophyll *a* (BChl) molecules and one carotenoid. The high resolution of this structure reveals all of the interpigment and pigment–protein interactions that promote the assembly and energy-transfer properties of this complex. Near the cytoplasmic face of the complex there is a ring of nine BChls, which absorb maximally at 800 nm and are designated as B800; each B800 is coordinated by the N-terminal carboxymethionine of LH2-α, part of a network of interactions with nearby residues on both LH2-α and LH2-β and with the carotenoid. Nine carotenoids, which are spheroidene in the strain we analyzed, snake through the complex, traversing the membrane and interacting with a ring of 18 BChls situated toward the periplasmic side of the complex. Hydrogen bonds with C-terminal aromatic residues modify the absorption of these pigments, which are red-shifted to 850 nm. Overlaps between the macrocycles of the B850 BChls ensure rapid transfer of excitation energy around this ring of pigments, which act as the donors of energy to neighboring LH2 and reaction center light-harvesting 1 (RC–LH1) complexes.

## Introduction

In many species of phototrophic bacteria, light-harvesting 2 (LH2) antenna complexes form a modular array of closely packed complexes for collecting solar energy. The intracytoplasmic membranes that house these arrays have been imaged by atomic force microscopy (AFM),^1,2^ showing the extent of the contacts made between LH2 complexes and in some cases the plasticity of the LH2 array in cells grown under high- or low-light intensity.^3,4^ In *Rhodobacter (Rba.) sphaeroides*, as with all of these bacteria, the LH2 antenna is the dominant photosystem component, which, along with reaction center light-harvesting 1 (RC–LH1), cytochrome *bc*_1_, and ATP synthase complexes, forms a bioenergetic network that converts absorbed solar energy to a chemical form, ATP.^5−7^ In *Rba. sphaeroides*, these four types of complex collectively assemble to form spherical intracytoplasmic vesicles, and whereas there are 200–300 vesicles per cell in cultures grown under high-light intensities, low-light cells can contain up to 1500 vesicles.^4^ Within each vesicle, approximately 60–70 LH2 complexes form LH2-only domains, the extent of which depends on the incident light intensity.^4^ Thus, the number of LH2 complexes per cell is under two levels of control.

The vesicles (“chromatophores”) of *Rba. sphaeroides* have been used to model the interlinked bioenergetic processes of excitation and electron transfers, formation of a protonmotive force, and its consumption by the ATP synthase.^5−7^ A structure of the *Rba. sphaeroides* LH2 complex would improve our understanding of LH2-enriched membrane domains in chromatophores,^8,9^ provide a molecular interpretation of previous site-directed modifications of the LH2 complex, and also underpin future manipulations of the pigment and protein components. Earlier structural studies of LH2 complexes used X-ray crystallography, and the first, iconic, structure was determined for the LH2 from *Rhodoblastus (Rbl.) acidophilus* (formerly *Rhodopseudomonas (Rps.) acidophila*) strain 10050 by Richard Cogdell and co-workers over 25 years ago.^10^ There was a subsequent improvement in resolution,^11^ and this LH2 structure was joined by others, from *Rhodospirillum molischianum,*(12)*Rbl. acidophilus* strain 7050,^13^ and *Ectothiorhodospira haloalkaliphila.*(14) A cryo-EM analysis of tubular, two-dimensional (2D), crystals of the *Rba. sphaeroides* LH2 complex was published in 1998;^15^ calculation of a projection map at a 6 Å resolution showed a ring of nine subunits, emphasizing the similarity in the overall architecture with the LH2 from *Rps. acidophila*. In the intervening time, and despite long-standing attempts by the present authors, and no doubt other research groups, to obtain highly diffracting crystals, there has been no high-resolution structure available for the *Rba. sphaeroides* LH2 complex.

Recently, there has been a remarkable upsurge in determinations of protein structures by single-particle cryo-EM,^16^ driven by a series of developments in sample and grid preparation, microscopes, detectors, and software.^17,18^ These advances offered new possibilities, circumventing the obstacles to crystallographic work presented by poorly ordered crystals. A series of papers reported the cryo-EM structures of RC–LH1 complexes from several purple phototrophic bacteria,^19−21^ and recently, the first cryo-EM structure of an LH2 complex, from *Marichromatium (Mch.) purpuratum*, was determined at a resolution of 2.4 Å.^22^ This LH2 comprises a ring of seven αβ heterodimer subunits, unlike the 8-fold and 9-fold rings from other bacteria reported earlier.^11,12,15^ Here, we present the cryo-EM structure of the *Rba. sphaeroides* LH2 complex at a 2.1 Å resolution, which reveals the intricate network of pigment and protein interactions that form the basis of its stability and its function as an antenna. The results are placed in the context of other LH2 structures^22,23^ and previous protein engineering studies of the *Rba. sphaeroides* LH2 complex.

## Materials and Methods

### Cell Culture

*Rba. sphaeroides* 2.4.1 mutant B7 (Δ*puc2BA* Δ*crtA*) was grown photosynthetically at 30 °C with stirring in 20 L M22+ medium under a light intensity of 150 μmol photons s^–1^ m^–2^ provided by Osram 116 W halogen bulbs. When the culture reached an absorption at 680 nm of 1.6, the cells were harvested by centrifugation at 3 000 *g* for 30 min and then washed once using working buffer (20 mM HEPES, pH 7.8). The pellet was stored at −80 °C until required.

### Protein Purification

Thawed cells were resuspended in working buffer with a few crystals of DNase and magnesium chloride, and then the suspension was passed through a French pressure cell three times at 18 000 psi. The broken cells were layered onto a 15/40% (w/w) sucrose density gradient. After 4 h of centrifugation in a Beckman SW32 rotor at 100 000 *g*, photosynthetic membranes were harvested from the interface, pelleted, then resuspended in working buffer, and adjusted to an absorbance of 100 at 875 nm. For solubilization, the 875 nm absorbance of membranes was 60, and the final concentration of *N*,*N*-dimethyldodecylamine *N*-oxide (LDAO) detergent was 3% (w/w). The mixture was stirred at 4 °C for 30 min, and then unsolubilized material was removed by centrifugation for 1 h at 211 000 *g* (Beckman 70.1 Ti rotor). The supernatant was applied to a five-layer (20/21.25/22.5/23.75/25% w/w) sucrose gradient in running buffer (working buffer containing 0.1% LDAO). After 16 h of centrifugation at 125 000 *g* in a Beckman SW41 rotor, the LH2 band, which was separated from the core RC–LH1–PufX complex, was collected and applied to an ion-exchange column (DEAE-Sepharose, Sigma-Aldrich) equilibrated with running buffer. Fractions containing the LH2 complex, which eluted at ∼150 mM NaCl, were collected, and those with an A_850/280_ absorption ratio of >2.7 were pooled and concentrated. LH2 was further purified on a gel filtration column (Superdex 200) pre-equilibrated in running buffer. Fractions with A_850/280_ > 3.3 were kept and concentrated to an A_850_ of 150 for cryo-EM data collection.

### Elemental Analysis

Ten microliters of purified LH2 complex (∼4 mg/mL) were cast onto the surface of a lacey carbon transmission electron microscopy (TEM) grid. The protein sample was dried in air under a 100 W lamp. As a control, a grid with the LH2 from *Rps. acidophila* (∼2.5 mg/mL) was prepared in the same way. Energy-dispersive X-ray spectrometry (EDXS) was performed on an FEI Tecnai Osiris S/TEM operated in STEM mode at 200 kV at room temperature. Data were acquired on a Bruker Super-X system comprising four EDX detectors. Spectra were acquired by integrating for 64 s on an area of several square microns to ensure good sampling with a current of ∼1 nA.

### Grid Preparation

Protein solution (2.5 μL, ∼4 mg/mL) was applied on a QuantiFoil R1.2/1.3 300 mesh Cu grid, which was glow-discharged for 60 s before use. An FEI Vitrobot MK IV was used for freezing the grid, using the following settings: chamber humidity 95%; chamber temperature 4 °C; blotting time 2.5 s; blotting force 3; and wait time 30 s. The grid was plunge-frozen into liquid ethane cooled by liquid nitrogen. It was then stored in liquid nitrogen until use.

### Cryo-EM Data Collection

Cryo-EM data were collected on a Thermo Fisher Titan Krios G2 cryogenic electron microscope equipped with a Gatan BioQuantum K3 direct electron detector at the Cambridge Pharmaceutical Cryo-EM Consortium.^24^ The microscope was operated at 300 kV with a normal magnification of 130 000x, corresponding to a pixel size of 0.66 Å at the specimen level. All movies were collected in super-resolution mode with an energy selecting slit of 20 eV. A total dose of 41.6 electrons per Å^2^ within a 1.37 s exposure time was fractionated into 40 frames, resulting in an electron fluence of 1.04 e^–^/Å^2^/frame. Automatic data collection was carried out in Thermo Fisher EPU 2.11 with two exposures per hole in aberration-free shift (AFIS) mode. In total, 3 138 movies were collected in a defocus range from −0.8 to −2.2 μm within 10 h.

### Data Processing

The initial super-resolution movie files were motion-corrected within RELION^25^ with a binning factor of 2 on 5 × 5 patches, resulting in a pixel size of 0.66 Å in the motion-corrected images. The contrast transfer function (CTF) parameters were estimated using CTFFIND4.^26^ All bad images, including those with empty holes, uncorrected drift, thick ice or extensive ice contamination were rejected. Particles were picked in cisTEM^27^ with a box size of 180 Å. In total, 1 717 608 particles were picked from all selected images, corresponding to an average of 547 particles per image. All extracted particles were subjected to reference-free 2D classification. After discarding bad 2D classes, 1 476 723 (86%) were selected for 3D classification. Reference-free 2D classification showed that the LH2 from *Rba. sphaeroides* is a nonamer (see Figure S1), which possesses an architecture similar to the LH2 from *Rbl. acidophilus*. A crystal structure model of this complex (PDB 1NKZ), therefore, was used to build an initial model for 3D classification using Chimera.^28^ Owing to the homogeneous subunits in the current LH2 complex, a C9 symmetry was imposed starting from the 3D classification. During the 3D classification, 835 641 (48.6%) particles were grouped into the best class (3.6 Å) out of 4. After multiple rounds of 3D refinement, CTF refinement (including anisotropic magnification, beam tilt, trefoil, fourth-order aberration, defocus estimation per particle, and astigmatism estimation per image) and Bayesian polishing with the default parameter in RELION 3.1, this data set produced a 2.3 Å resolution map. The selected particles were re-extracted using 25 nm × 25 nm box for CTF refinement and further Bayesian polishing. This resulted in a final 3D map with a resolution of 2.1 Å.

### Refinement and Modeling

**Figure 1:**
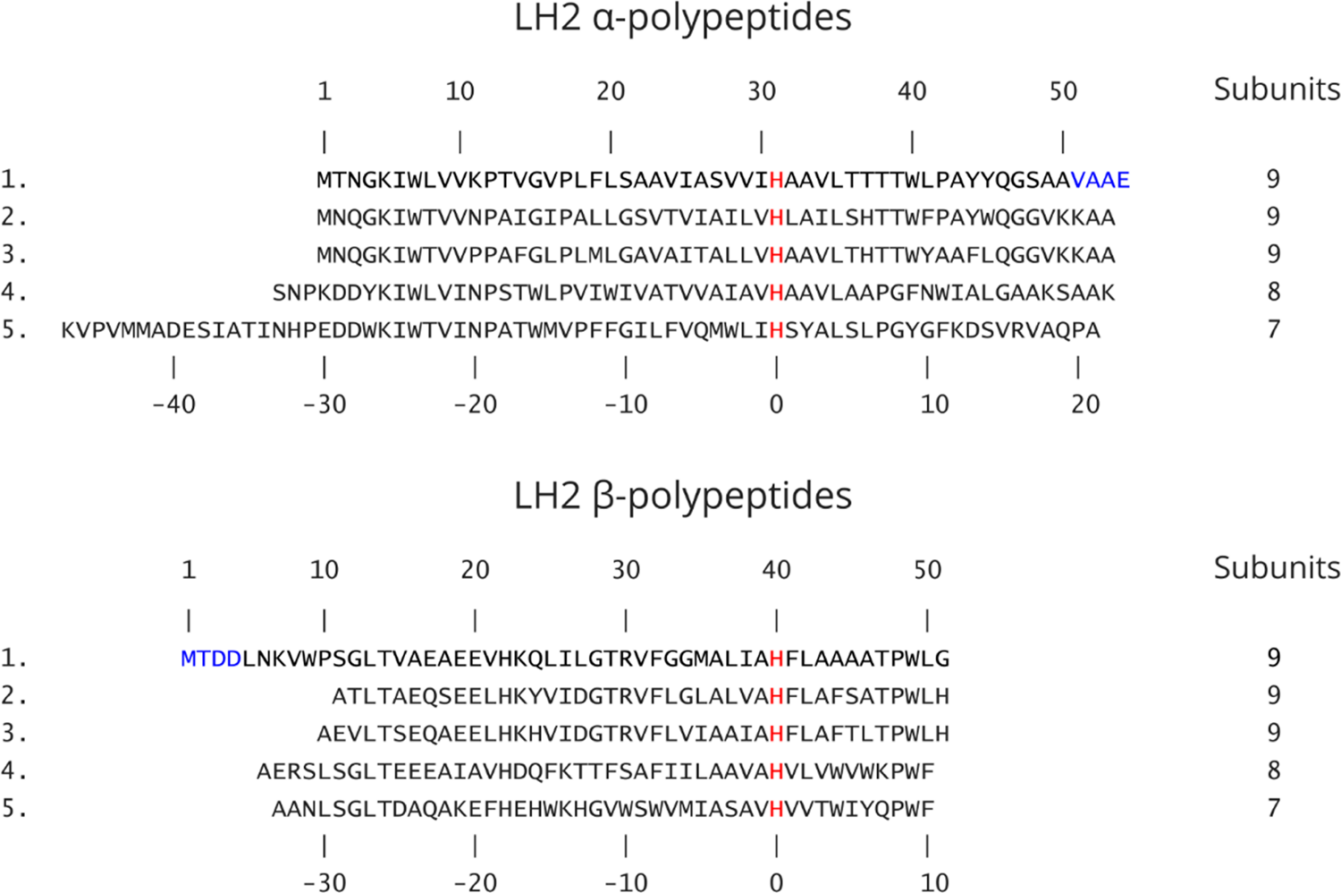
Amino acid sequence alignments of α- and β-polypeptides in LH2 complexes for which a high-resolution 3D structure is available. 1, *Rba. sphaeroides* 2.4.1 (PDB 7PBW); 2, *Rbl. acidophilus* 10050 (PDB 1NKZ); 3, *Rbl. acidophilus* 7050 (PDB 1IJD); 4, *Phs. molischianum* DSM-119 (PDB 1LGH); and 5, *Mch. purpuratum* DSM-1591 (PDB 6ZXA). The sequences shown in black are those reported and resolved in the PDB entries; the blue characters in the α and β sequences for *Rba. sphaeroides* indicate those not modeled into the density map. The sequences are aligned against the central His residue (red) that coordinates the BChl *a* molecules forming the main ring of pigments. The top set of numbers refers to the residues in the *Rba. sphaeroides* LH2 complex. The lower set of numbers designates the His ligand to the B850-type BChls (red) as 0 and counts forwards or backwards from that point. This numbering system, from a central, conserved residue, allows comparison of polypeptides of differing lengths and from different bacteria. The LH2 oligomer sizes (number of αβ subunits) for different strains are indicated.

An α/β subunit having three BChl *a* and one carotenoid taken from LH2 of *Rbl. acidophilus* (PDB 1NKZ) was docked into the C9 symmetric density map of LH2 from *Rba. sphaeroides* using Chimera, focusing on correct positioning of the B850 pairs and their coordinating His residues. Amino acid sequences of both α/β polypeptides in the template were mutated in COOT^29^ based on the *Rba. sphaeroides* sequences in Figure 1. The carotenoid in the template was replaced by spheroidene, and its orientation was decided according to its head group densities. Thus, a subunit of LH2 from *Rba. sphaeroides*, αβBChl_3_Car, was constructed. After being real-space-refined in COOT, all symmetry copies were generated to form a preliminary model of the complete LH2 complex. A preliminary refinement was performed in COOT, and tightly bound detergent molecules, LDAO, were added. The model was then rebuilt and optimized in ISOLDE,^17^ including the addition of 230 water molecules and a calcium ion coordinating the final turn of each β chain helix. The rebuilt model was subjected to restrained global refinement in Phenix^30^ using Phenix real_space_refine. The refinement statistics are summarized in Table S1. The refined model and its map were deposited into Protein Data Bank (PDB) and the Electron Microscopy Data Bank (EMDB) with access codes of 7PBW and EMD-13307, respectively.

## Results

### Overall Structure of the *Rba. sphaeroides* LH2 Complex

**Figure 2:**
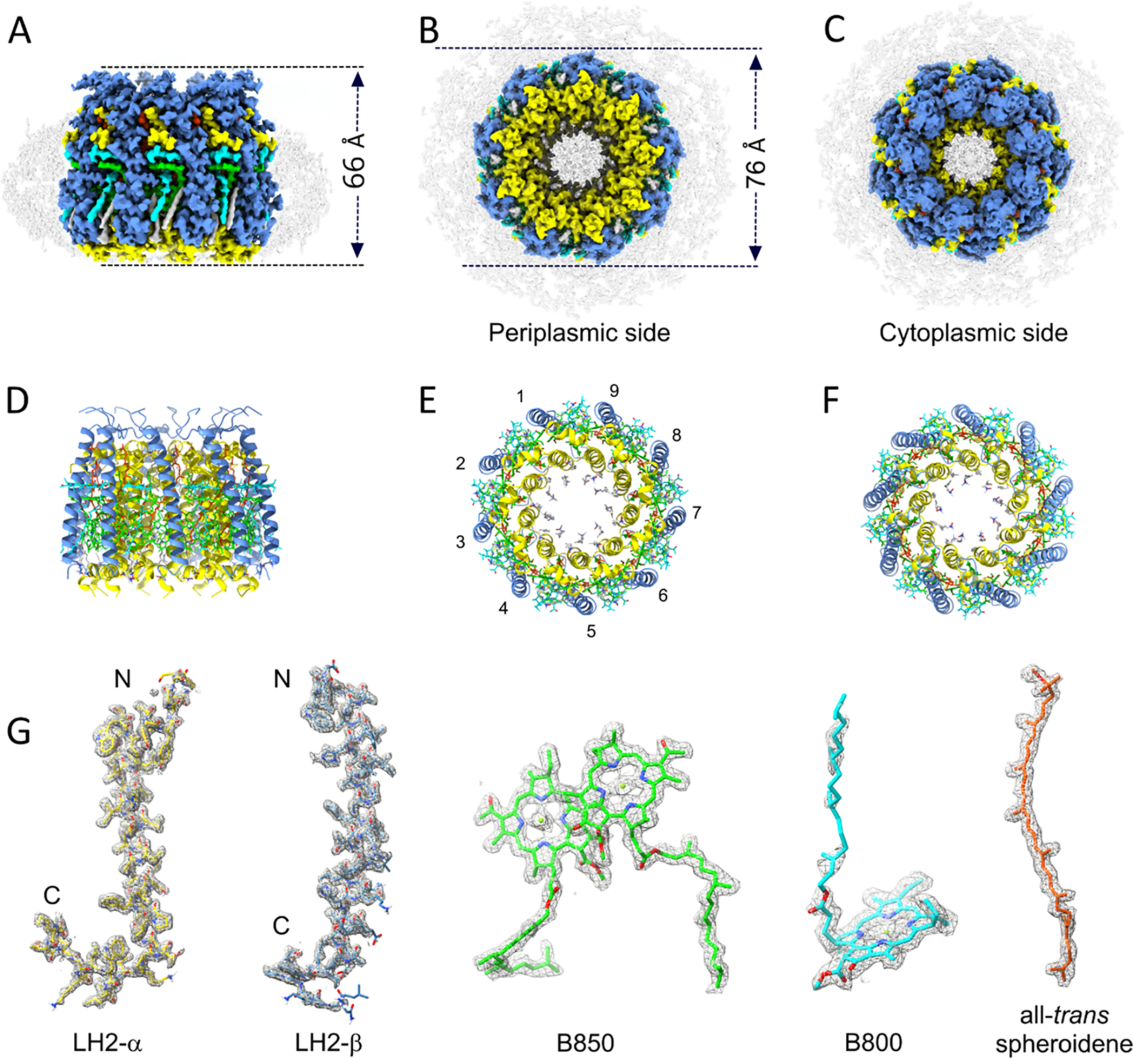
Cryo-EM structure of the LH2 from *Rba. sphaeroides*. (A–C) Cryo-EM map of the LH2 complex viewed in the plane of the membrane and from the periplasmic and cytoplasmic sides, respectively. The dimensions of the complex are shown in panels (A) and (B). The complex is surrounded by a belt (in gray) of disordered densities composed of detergents and lipids. (D–F) The LH2 complex in ribbon format, with the same orientations as in the top row and with the nine αβ heterodimers numbered in panel (E). (G) Fits of LH2 α- and β-polypeptides, a B850 pair of BChls, a B800 BChl, and a spheroidene carotenoid within their respective densities, with the maps at 9 sigma. N and C indicate the N- and C-termini, respectively. Color code: α-polypeptide, *yellow*; β-polypeptide, *medium blue*; B850, *green*; B800, *cyan*; spheroidene, *orange*; and detergent belt, *gray*.

The LH2 complex was purified from *Rba. sphaeroides* strain B7, which was engineered to ensure uniformity of the complexes assembled. First, the *puc*2*BA* genes encoding the second set of LH2 αβ polypeptides^31^ were removed by in-frame deletion, leaving only the *puc*1*BAC* operon to provide polypeptides for the LH2 complex and with PucC as an assembly factor.^32^ Second, a *crtA* mutation was used to inactivate spheroidene monooxygenase (CrtA), which introduces a C2 keto group into the yellow spheroidene to form the red carotenoid spheroidenone.^33^ Restricting the carotenoid pathway in this way removes the possibility of LH2 complexes assembling with a mixture of spheroidene and spheroidenone, minimizing heterogeneity. We recorded 3 138 cryo-EM movies, from which 1 717 608 particles were picked for further data processing, yielding a final resolution of 2.1 Å (Figures 2 and S1 and Table S1). Statistics for the final model are in Table S1. The 2.1 Å resolution allows nearly all of the α- and β-polypeptides to be modeled into the density (Figure 2), and the central Mg atoms of the BChls can be assigned, as well as the terminal methoxy and seven methyl groups for spheroidene. Figure 2A–C shows the overall size and shape of the complex, which is 66 Å in height (Figure 2A) and 76 Å in diameter (Figure 2B). The belt of density (in gray) that surrounds the complex (Figure 2B–D) arises from the micelle of LDAO detergent molecules used to solubilize the complex and likely also from some phospholipids retained from the native membrane. Figure 1D–F shows the model of the LH2 complex with the α- and β-polypeptides in ribbon representation. The LH2 complex is assembled as an inner ring comprising nine transmembrane α-apoproteins, which is 46 Å diameter in the transmembrane part of the complex and an outer, 76 Å diameter ring of nine β-polypeptides. The central hole has a diameter of 20 Å on the cytoplasmic side of the complex and 27 Å on the periplasmic side and consists of disordered densities arising from detergent and lipid, but only lipids are expected to be present in the LH2 embedded in the native membrane. The α- and β-polypeptides have a central α-helical domain flanked by N- and C-terminal regions that lie close to the cytoplasmic or periplasmic membrane surface, respectively, although the N-terminal loop of β extends from the cytoplasmic surface. Figure 1G shows the polypeptide, bacteriochlorophyll (BChl), and carotenoid components of LH2 docked within their respective densities taken from the final refined model.

### Protein–Protein and Pigment–Protein Interactions That Stabilize the LH2 Ring

**Figure 3:**
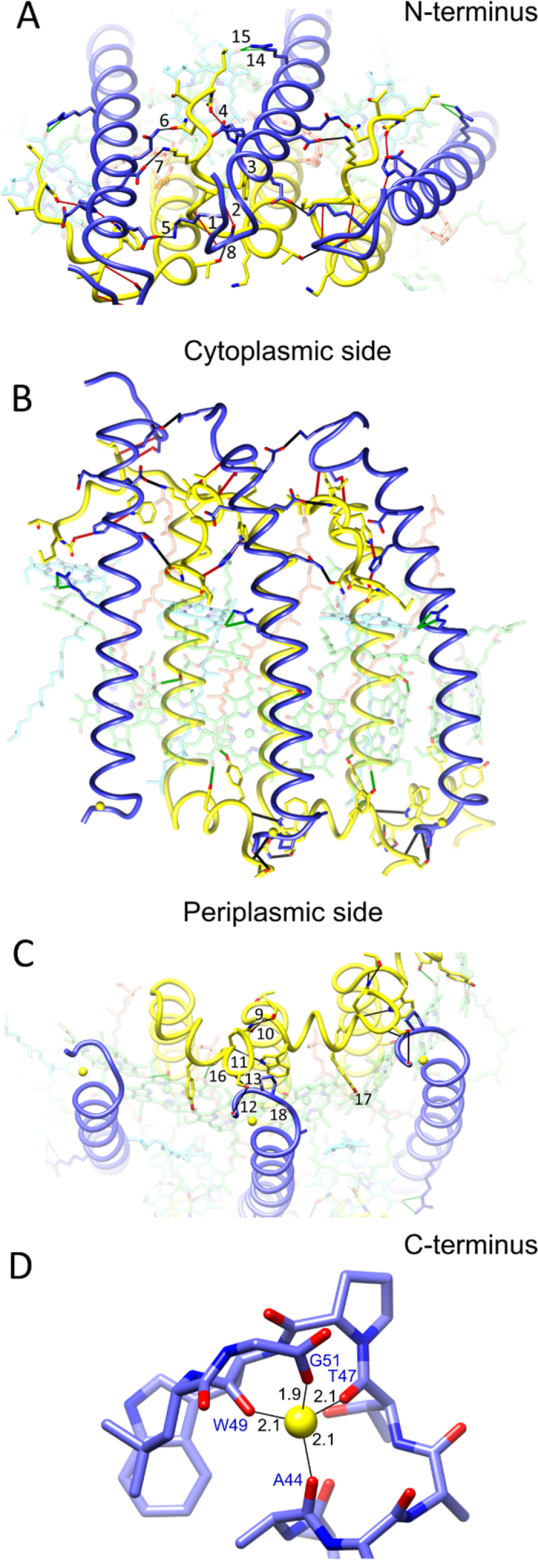
Protein–protein and pigment–protein interactions in the *Rba. sphaeroides* LH2 complex. (A) N-terminal regions of three αβ heterodimer subunits viewed from the cytoplasmic side of the complex, with hydrogen bonds numbered according to Table 1 and with intra-subunit bonds in red, inter-subunit bonds in black, and protein–ligand bonds in green. To emphasize the polypeptides, the pigments are semitransparent. (B) Three αβ heterodimer subunits viewed in the plane of the membrane. (C) C-terminal regions of three αβ heterodimer subunits viewed from the periplasmic side of the complex, with hydrogen bonds numbered and colored as in panel (A). Three Ca^2+^ ions are shown as yellow spheres. (D) Coordination of the Ca^2+^ by C-terminal residues labeled in blue, and the bond lengths are indicated. The color code is the same as in Figure 1: α-polypeptide, *yellow*; β-polypeptide, *blue*; B850, *green*; B800, *cyan*; and carotenoid, *orange*.

The LH2 structure consists of nine repeating αβ polypeptide subunits, three of which are shown in Figure 3A–C. LH2α consists of a short N-terminal helix of 12 residues that lies on the surface of the membrane on the cytoplasmic side, followed by a 25-residue transmembrane domain and a short C-terminal region comprising 13 residues. Similarly, the LH2 β-polypeptide has N-terminal, transmembrane, and C-terminal regions of 14, 32, and 5 residues, respectively. An extensive network of hydrogen bonds stabilizes the LH2 structure (Figure 3A–C); all of the intra-heterodimer bonds are between N-terminal regions (Figure 3A,B), whereas bonds between LH2 αβ subunits are on both sides of the complex (Figure 3A–C). There are no hydrogen bonds between the transmembrane sections of LH2 α and β polypeptides. Table 1 presents a full list of the hydrogen bonds found in the LH2 structure, and the same numbering is used in Figure 3A,C, also with color-coding to identify intra- (red) and inter- (black) heterodimer bonds, as well as protein–ligand bonds in green.

**Table 1:** Hydrogen Bonds within and between LH2 αβ Heterodimers^a^,^b^

number	residue 1	atom 1	residue 2	atom 2	distance (Å)
**intra-αβ heterodimer**					
1	β-Ser11 (bb)	N	α-Leu8 (bb)	O	2.88
2	β-Ser11 (side)	OG	α-Trp7 (bb)	O	2.42
3	α-Trp7 (side)	NE1	β-His22 (side)	ND1	2.97
4	β-His22 (side)	NE2	α-COO-Met1 (nt)	ON1	2.54
**inter-αβ heterodimer**					
5	β-Lys7 (side)	NZ	β-Glu17 (side)	OE2	2.92
6	β-Gln24 (side)	NE2	α-Asn3 (side)	OD1	2.97
7	α-Lys5 (side)	NZ	β-Glu20 (side)	OE2	3.05
8	α-Lys11 (bb)	N	α-Thr13 (side)	OG1	3.17
9	α-Gln46 (side)	NE2	α-Thr39 (side)	OG1	2.69
10	α-Thr39 (bb)	N	α-Gln46 (side)	OE1	2.92
11	α -Trp40 (side)	NE1	α-Tyr45 (bb)	O	2.93
12	α-Ser 48 (side)	OG	β-Gly51 (ct)	OXT	2.77
13	α-Ser 48 (bb)	N	β-Pro48 (bb)	O	3.10
**protein–ligand**					
14	β-Arg30 (side)	NE	α-BCL1702	OBB	2.96
15	β-Arg30 (side)	NH2	α-BCL1702	OBB	3.04
16	α-Tyr45 (side)	OH	α-BCL1502	OBB	2.51
17	α-Tyr44 (side)	OH	β-BCL1602	OBB	2.57
18	α-Ser27 (side)	OG	β-BCL1602	OBD	2.74

a The numbers correspond to the labels in Figure 3. The atom labels refer to those used in the accompanying structure file (PDB: 7PBW). The BCL numbers specify the BChls bound to the LH2 α and β polypeptides. BCL1702 is the B800 BChl; BCL1502 and BCL1602 are B850 BChls.

b bb, backbone; side, side chain; nt, N-terminus; and ct, C-terminus.

Figure 3C shows that the C-terminus of each β-polypeptide binds a calcium ion, shown in more detail in Figure 3D. Energy-dispersive X-ray analysis (EDX) was used for qualitative analysis of metal ions in LH2 complexes from *Rba. sphaeroides*, with the *Rbl. acidophilus* LH2 as a control (Figure S2). Whereas bound Mg^2+^ was found in the *Rbl. acidophilus* LH2, EDX identified Mg^2+^ in the *Rba. sphaeroides* complex and new peaks appeared for Ca^2+^. Based on this result combined with the coordination bond lengths and geometry, we conclude that it is reasonable to assign the C-terminal metal as calcium. This metal is held to the C-terminus of the LH2 β-polypeptide by the three backbone oxygens of β-Ala44, Thr47, and Trp49 and by the C-terminal carboxylate of Gly51. We found some evidence in the density map for two water molecules coordinating with the Ca (bond lengths 3.2 and 2.7 Å; not shown), giving an approximate octahedral geometry, but they are not assigned in the final model. The role of these metals is unclear; in one treatment, we incubated the *Rba. sphaeroides* complex with excess EDTA to remove all bound ions, in another the complex was fully reconstituted with excess CaCl_2_, and a third treatment used EGTA and excess MgCl_2_ to strip the endogenous metal and replace it with Mg^2+^. None of these procedures affected the absorption spectrum of the complex (Figure S3).

### Packing of the Bacteriochlorophyll and Carotenoid Pigments within the LH2 Complex

**Figure 4:**
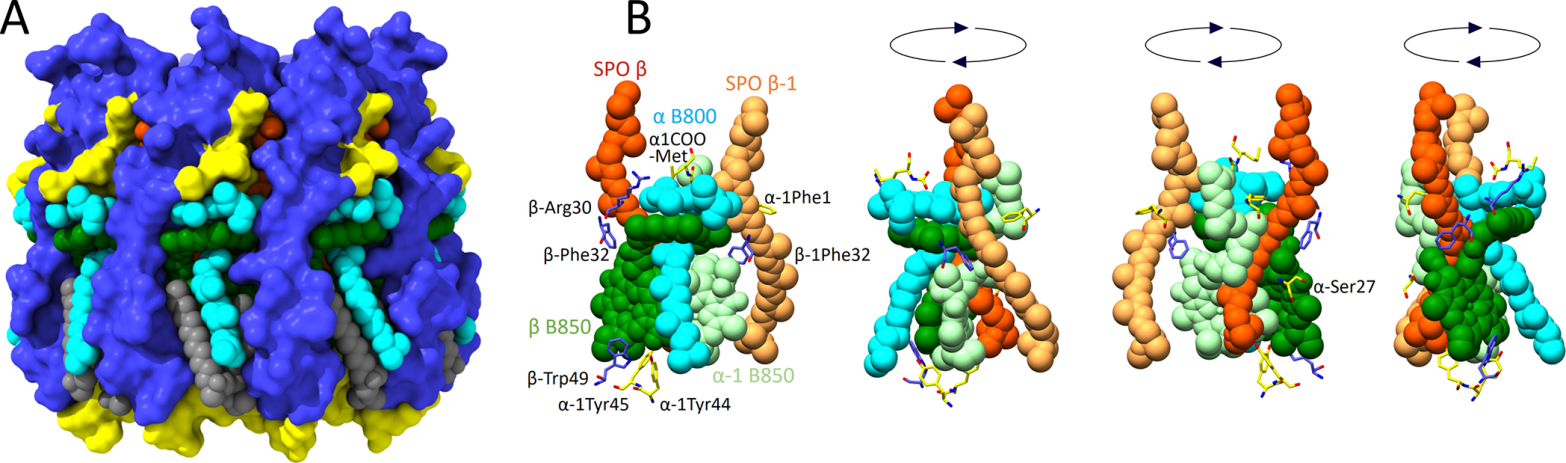
Tight packing of pigments within the LH2 complex. (A) Complete complex viewed in the plane of the membrane with the polypeptides in surface representation and pigments in a spacefill representation with the cytoplasmic side uppermost, showing the outer edge of the B800 BChls (cyan), part of the B850 phytyl tail, and LDAO detergents (gray) packed against the B800 phytyls. (B) Compact unit of B800 (cyan), B850 (green), and carotenoid (orange) pigments showing interactions between pigments in adjacent αβ heterodimers denoted by green/pale green and orange/pale orange. Nearby residues that interact with these pigments are shown in the first image, with successive 90° rotations used for the other images.

Figure 4A shows a surface representation of the LH2 polypeptides, with pigments in a spacefill representation. Rings A and B of the B800 BChls are exposed to the lipid bilayer, with the phytyl tails running down the side of LH2-β and also packed against a detergent molecule (LDAO; gray). The end of each B850 phytyl tail (green) wraps around the lower face of the B800 macrocycle. Removal of the LH2 α and β polypeptides reveals the extent of the contacts between pigments, with the phytyl tail of B850 (green) forming a hydrophobic interaction with the lower face of B800, seen from several angles in Figure 4B. Ring C/E of the B800 (cyan) contacts C18–C22 (counting from the carotenoid ether group next to B850; see Figure S4) of the carotenoid from the adjacent αβ heterodimer (pale orange) and the C8–C12 region of spheroidene in darker orange makes a series of hydrophobic contacts with ring D of the α-bound B850 (pale green) in the adjacent αβ heterodimer. The overall effect is a circular chain of carotenoid interactions interlinking each αβ heterodimer with its neighbor. The extent of the contacts between the carotenoid and surrounding pigments and proteins accounts for the decisive contribution made by this pigment to the stability of LH2.^34−36^Figure 4B also shows how mainly hydrophobic side chains pack against the carotenoids and help to stabilize them within the complex.

### Overall Organization of Bacteriochlorophyll and Carotenoid Pigments

**Figure 5:**
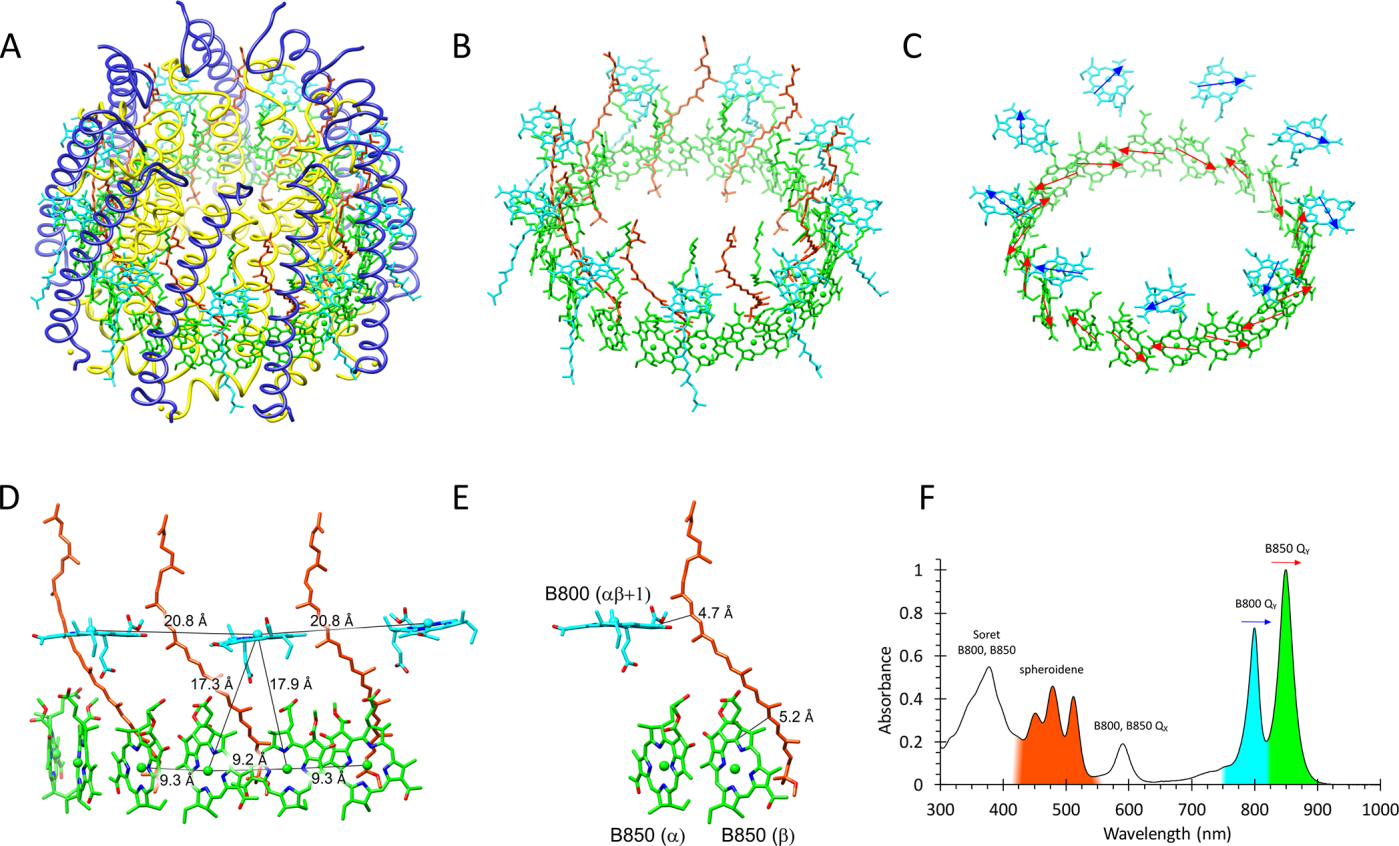
Bacteriochlorophyll and carotenoids in the *Rba. sphaeroides* LH2 complex. (A) Tilted view of the complete complex. (B) The same view as in panel (A), but with the protein components removed to show the rings of B800 (cyan), B850 (green), and carotenoid (spheroidene, red) pigments. (C) The same view as in panel (B) but with the BChl phytyls removed for clarity and arrows (blue for B800, red for B850) indicating the absorption transition dipoles. (D) Pigments that bind to three αβ heterodimer subunits (polypeptides not shown) viewed in the plane of the membrane showing the Mg–Mg distances between BChls. (*E*) A subset of pigments from panel (D) showing the closest approach of a spheroidene molecule to a B800 from the adjacent αβ heterodimer and a B850 BChl. (F) Absorbance spectrum of the purified LH2 complex, with some absorbance bands color-coded according to the LH2 pigments involved. Other features such as the Soret and Q_X_ bands consist of contributions from both rings of BChl pigments and are not colored. Colors as in Figure 1: α-polypeptide, *yellow*; β-polypeptide, *blue*; B850, *green*; B800, *cyan*; and carotenoid, *orange*.

Figure 5A shows the complete LH2 structure with all polypeptide and pigment components, and in Figure 5B, the LH2 α and β polypeptides have been removed to reveal the pigment rings of nine B800 BChls, nine carotenoids, and 18 B850 BChls. The orientation of the B800 BChls and their Q_Y_ absorption transition dipoles are almost parallel to the plane of the membrane, in approximate alignment with the head-to-tail Q_Y_ transitions of the paired B850 BChls (Figure 5C), which are oriented with their macrocycles perpendicular to the plane of the membrane. The clear separation between B800 BChls is in marked contrast to the overlapping macrocycles of the B850 BChls, which accounts, in part, for the differing absorption properties of these rings of BChls shown in Figure 5F. To simplify this network of interacting chromophores, Figure 5D shows a subset of pigments that bind to three αβ heterodimer subunits (polypeptides not shown), viewed in the plane of the membrane. The spacing of B800 BChls is 20.8 Å (Mg–Mg), whereas the alternating 9.3 and 9.2 Å Mg–Mg distances for B850 BChls reflect intra- and interdimer pairs, respectively. The alignments and close spacing of BChls create the conditions for the fast, 0.7 ps, transfer of excitation energy from B800 to B850.^37^Figure 5F shows how the various pigments contribute to the absorption of the complex, notably the carotenoid spheroidene in the 420–540 visible region, and the Q_Y_ bands for B800 and B850 in the 750−900 nm near-infrared region of the spectrum.

Figures 4 and 5 show that the carotenoid spheroidene adopts an all-trans conformation as it winds through the LH2 complex, making a series of contacts with the B800 macrocycle, as specified in the previous section. The carotenoid passes within 4.7 Å (Crt C20) and 5.2 Å (Crt C8) of the nearest B800 and B850 BChl, respectively (Figure 5E). These van der Waals contacts are consistent with the short excited-state lifetime of ∼1.6 ps for spheroidene arising from ultrafast energy transfer to these BChls^38^ and the 96% efficiency of this process.^36^ To separate their functional and structural roles further, B800 and B850 will be dealt with separately.

### Pigment–Protein Interactions: The B800 Bacteriochlorophyll Binding Site

**Figure 6:**
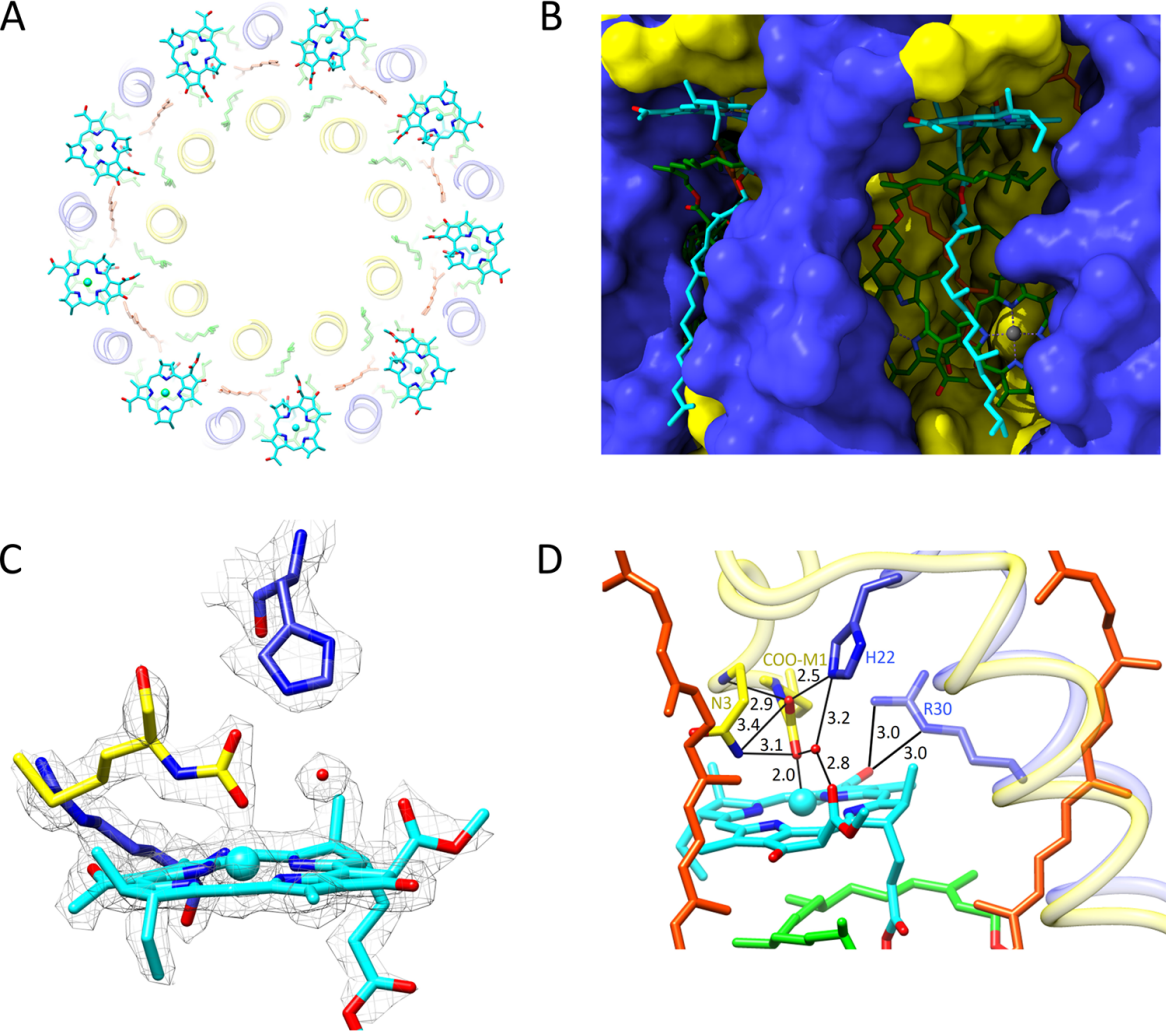
B800 BChls. (A) The ring of B800 molecules, with Mg–Mg distances of 20.8 Å (Figure 5D), viewed from the cytoplasmic side of the complex. (B) View of the B800 binding site from the exterior of the complex in the plane of the membrane. The polypeptides are in surface representation with the cytoplasmic side uppermost, showing the outer edge of the B800 BChls (cyan), part of the B850 macrocycle and the phytyl tail (green). (C) Fits of a B800 (cyan), carboxymethyl methionine (α-Met1) (yellow), β-His22 (blue), and a water molecule (red sphere) modeled within the density in this region of the complex. (D) Structural model of the B800 binding site showing distances of hydrogen bonds and the coordination with the Mg of B800. β-His22 sits above B800, and β-Arg30 is on the right forming hydrogen bonds with the B800 BChl. The bonding network includes α-Asn3. Two carotenoids are shown, one of which passes close to the B800.

The 9 B800 BChls are well spaced in comparison with the 18 B850 BChls (Figure 6A). Each B800 sits in a pocket approximately 10 Å from the interface between the external solvent and the cytoplasmic membrane (Figure 6B), held in place by many interactions, including one with a water molecule, which is well resolved in the density map (Figure 6C). The model in Figure 6D shows that this water molecule occupies a central role by bonding with the C13^2^ ester oxygen of B800, while being held in place by hydrogen bonds with β-His22, and one of the α-COO-Met1 carboxyl oxygens. The N-terminal carboxyl also bonds with β-His22, distal from the B800, and on the proximal side, the other carboxyl oxygen forms a hydrogen bond to α-Asn3 and a ligand to the B800 Mg. This type of ligand was also observed in the LH2 from *Rps. acidophila* strain 10 050 refined to 2.0 Å resolution.^11^ This extensive bonding network appears to hold the *N*-carboxyl of the methionine in position so it can form a ligand to B800. The participation of β-His22 is important, and mutagenesis to serine removed almost all of the B800 absorption.^39^ One interaction of particular interest, first identified by site-directed mutagenesis,^39−41^ is with β-Arg30, which exerts a strong influence on B800 and also on the nearby carotenoid. The C3-acetyl carbonyl of B800 forms hydrogen bonds with the β-Arg30 side chain, as shown by an earlier resonance Raman and mutagenesis study.^40^ This arrangement of hydrogen bonds distorts the B800 slightly, and the C3-acetyl group is rotated about 25° out of plane toward β-Arg30.

### Pigment–Protein Interactions: The B850 Binding Sites

**Figure 7:**
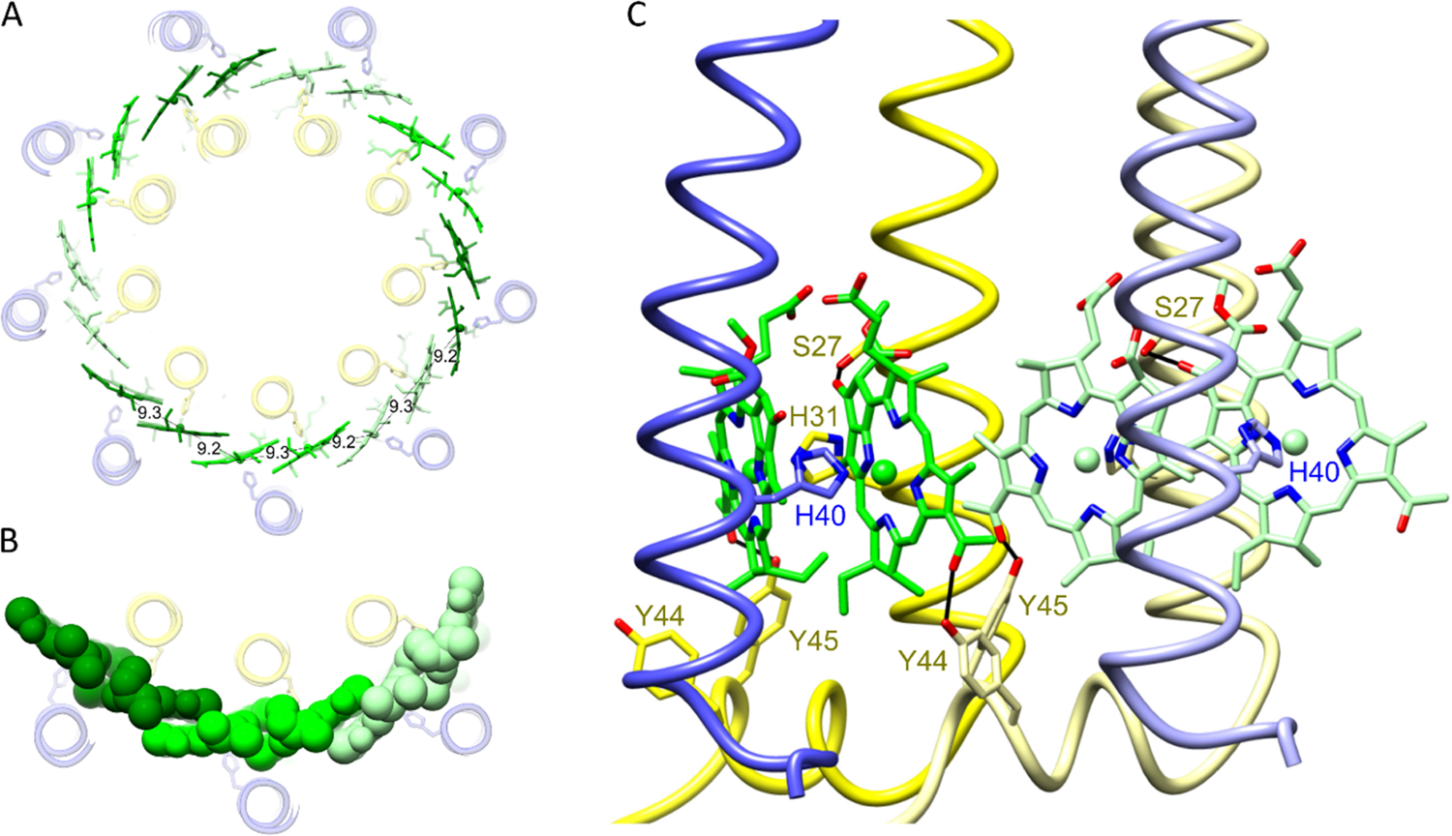
B850 BChls. (A) The ring of 18 B850 molecules viewed from the periplasmic side of the complex, with Mg–Mg distances of 9.2 and 9.3 Å shown. (B) Three pairs of B850 BChls in a spacefill representation. (C) Structural model of two adjacent B850 dimer binding sites showing hydrogen bonds between LH2 α-Tyr44, α-Tyr45, and a C3-acetyl carbonyl on each BChl. α-Ser27 is hydrogen-bonded to the C13^1^ keto on ring E. Different shades differentiate the αβ heterodimers, with the α-polypeptide in *yellow*; β-polypeptide, *blue*; and B850, *green*. B800, phytyl tails, and carotenoids have been removed for clarity.

Toward the periplasmic side of the LH2 complex, and ligated to α-His31 and β-His40, there are nine pairs of BChls, with their macrocycles oriented perpendicular to the plane of the membrane (Figure 7). The Mg–Mg distance between paired BChls within an αβ heterodimer is 9.3 Å, representing an overlap of pyrrole rings C and E (Figures 5D and 7A). There is a similar degree of overlap between rings A of B850s from neighboring heterodimers, given the 9.2 Å Mg–Mg distance. It is somewhat counterintuitive that the B850 BChls within an αβ heterodimer are further apart, albeit only slightly, than BChls in adjacent heterodimers, but the same was observed for the *Rbl. acidophilus* LH2 and LH3 complexes, both with intradimer and interdimer Mg–Mg distances of 9.5 and 9.0 Å, respectively.^10,13^Figure 7B shows a spacefill representation of three pairs of B850 BChls, illustrating the close packing of the B850 BChls that establishes continuous contact around the B850 ring, promoting intradimer and interdimer exciton coupling and red-shifting the absorption of these pigments with respect to the monomeric B800 BChls. A further red shift is imparted by hydrogen bonding between the LH2 α-Tyr45 and the C3-acetyl group on the B850 BChl bound to the LH2 α polypeptide in the same αβ heterodimer. The neighboring residue, LH2 α-Tyr44, also forms a hydrogen bond but to the C3-acetyl group on the BChl bound to the LH2 α polypeptide belonging to the next αβ unit in the ring (Figure 7C). These bonds shift the absorption of this pair of BChls to 850 nm, and it was shown many years ago that this effect can be reversed by mutagenesis of LH2 α-Tyr44 and Tyr45 to Phe and Leu, respectively, producing a B800–826 complex.^42,43^ From a structural and assembly point of view, this hydrogen-bonding pattern links a given αβ unit to the next one in the ring. We identified another hydrogen bond between α-Ser27 and the α-bound B850 (Figure 7C), which had been assigned earlier on the basis of a mutagenesis and resonance Raman study; alteration to alanine had no effect on the absorption maximum.^44^ This hydrogen bond is part of a series of interactions that impart stability to the LH2 complex, along with those involving carotenoids, phytyl tails, ligands to BChls, and hydrogen bonds. The B850 BChls have structural and energetic continuity, forming an approximately circular path for excitons and creating the conditions for ultrafast excitation transfer. Thus, the LH2 complex is ideally configured to transfer energy internally and to donate energy to neighboring LH2 and RC–LH1–PufX complexes.^45−48^

## Discussion

### B800 Bacteriochlorophylls

The *Rba. sphaeroides* LH2 antenna has been used extensively as a model for bacterial light-harvesting complexes, and indeed, this was the first such complex to be purified by Betty and Rod Clayton in 1972.^49^ They solubilized photosynthetic membranes with lauryl dimethyl amine oxide (LDAO), the same detergent successfully used by Rod Clayton and George Feher for isolating reaction center complexes.^50,51^ The other antenna complex in the *Rba. sphaeroides* photosystem, LH1, was destroyed by LDAO and its purification awaited more gentle solubilization by lithium dodecyl sulfate.^52^ This detergent had an interesting effect on LH2 by selectively removing nearly all of the B800 absorption band, which could be restored by the subsequent addition of LDAO.^53^ The structure of the *Rba. sphaeroides* LH2 complex provides a basis for these early observations and also for many subsequent pigment exchange studies that replaced B800 with BChl and chlorophyll (Chl) analogues.^54−56^ B800 sits within 10 Å of the solvent/membrane interface, sufficiently close to make it selectively susceptible to some detergents. Despite the network of hydrogen bonds between B800 and LH2 α and β polypeptides, and the interaction with the carotenoid spheroidene (Figures 3–5), pigments as diverse as 3-acetyl Chl *a*, Chl *d*, Chl *f*, or BChl *b* can substitute for B800.^55^ The provision of a hydrogen bond to the 3-acetyl group of native and non-native pigments by β-Arg30 was shown to be important for pigment exchange.^55^ This work and earlier studies referred to this residue as β Arg–10, employing a method of aligning LH sequences using the central His (His0) that coordinates the B850 BChls; counting forwards or backwards from that point allows comparison of LH polypeptides of differing lengths (see Figure 1, which shows both numbering systems). An earlier resonance Raman study of *Rba. sphaeroides* LH2 mutants in which β-Arg30 (β Arg–10) was altered to Phe, Leu, Glu, and Met^41^ examined the basis for the red-shifted B800 absorption of monomeric BChls bound within LH2 antenna complexes, in relation to the 771 nm absorption of BChl in ether.^40^ It was concluded that the hydrogen bond from β-Arg30 to the C3-acetyl group of the B800 BChl imparts a shift of approximately 10 nm, and another 10 nm of shift arises from the local properties of the BChl binding site.^40^ The structure in this region of the complex (Figure 6) puts some detail on the locality of B800, and at least some of the 10 nm red shift relative to monomeric BChl in solvent likely arises from the proximity of B800 to β-Arg30 and the carotenoid, from the local bonding network (Figure 6D), and from the ligand to the carboxymethionine at the N-terminus of LH2α. We note that a mass spectrometry study of the *Rba. sphaeroides* LH2 complex found no carboxylation modification of α-Met1,^57^ although it does report the N-carboxylation of Thr2 on the Puc2B polypeptide, which is not present in our complex. Thus, there is evidence that the mechanism for *N*-carboxylation exists in *Rba. sphaeroides*, supporting the feasibility of this modification also occurring on an N-terminal Met1. The density map in Figure 6C clearly supports the assignment of a carboxyl on α-Met1, and it shows how this carboxyl participates in a bonding network that involves β-His22, explaining our previous mutagenesis results in which alteration to serine removed almost all of the B800 absorption.^39^ The paper on the original structure of the *Rbl. acidophilus* LH2 complex determined by X-ray crystallography also assigned a carboxymethionine at the N-terminus of LH2α.^10^ In the vicinity of this carboxymethionine, we also found a clear density for a water molecule, which appears to play a pivotal role in the B800 binding site, given its interactions with protein side chains and with B800. McDermott et al. also reported a water molecule in the corresponding location near B800 of the *Rbl. acidophilus* LH2 complex.^10^

### LH2 Carotenoids

Related to the previous section, the LH2 structure emphasizes the central location of β-Arg30 and its proximity to the carotenoid. This residue senses changes in electrical potential across the membrane bilayer, which are relayed to the carotenoid, which responds with a shift in absorption.^58^ Such responses to illumination of whole cells were recorded over 60 years ago (e.g.,^59^) and subsequently calibrated against the membrane potential by Baz Jackson and Tony Crofts in 1969^60^ and later by many workers (e.g.^61^). Then, it became clear that these absorption shifts originated in the carotenoids bound to LH2,^62,63^ and a subsequent mutagenesis study of the *Rba. sphaeroides* LH2 complex showed that alteration of β-Arg30 greatly attenuated the carotenoid absorption shift that responds to the membrane potential.^57^ Ultrafast carotenoid band shifts, attributed to a local electric field have been observed for the LH2 complex following excitation of either B800 or B850 BChls;^64,65^ these band shifts might also stem from the presence of β Arg30. While spanning the membrane, the nine carotenoids make multiple contacts with protein side chains and with B800 and B850 BChls that stabilize the complex (Figures 5 and 6), to the extent that no LH2 assembly is possible in their absence.^34,35^ Despite these multiple contacts, which interlink successive αβ units in the nonamer ring (Figures 4 and 5), it is interesting to note that the *Rba. sphaeroides* LH2 not only tolerates a variety of native carotenoids such as neurosporene, spheroidene, and spheroidenone but also incorporates the non-native zeta-carotene,^66^ lycopene,^67^ as well as rhodopin, spirilloxanthin, and 2,2′-diketo-spirilloxanthin.^36^

### B850 Bacteriochlorophylls

The 850 nm absorption band of the *Rba. sphaeroides* LH2 arises from the ring of 18 overlapping BChls, which are held in a wider circle than the B828 BChls in the heptameric *Mch. purpuratum* ring.^22^ The larger, nonameric ring decreases the angle between BChl pairs, which slightly shortens the distance between the nearest Mg^2+^ atoms in adjacent αβ heterodimers, strengthening the exciton coupling around the ring and producing a 22 nm red shift from 828 nm in *Mch. purpuratum* to 850 nm in *Rba. sphaeroides*. Despite the many similarities between the present LH2 structure from *Rba. sphaeroides* and the nonameric LH2 from *Rbl. acidophilus* 10050, there is a small difference in absorption, with the ring of 18 BChls giving an 850 nm maximum in the former case and 858 nm in the latter. There is no obvious way to account for this difference, and given the large red shifts that arise from hydrogen bonding to the C3-acetyl carbonyls,^42^ possibly the most straightforward explanation could lie in small differences in hydrogen bond strength and in the angles made between these C3 acetyls and the plane of the BChl macrocycle. Natural variation has produced a nonameric complex termed LH3, with an absorption band at 820 nm; the structure of this antenna complex (PDB 1IJD) from low-light-grown *Rps. acidophila* strain 7050 has Phe and Leu at locations on the C-terminal region of LH2α where aromatic residues would normally form hydrogen bonds to B850 BChls (Figure 7C). In the case of *Rba. sphaeroides*, mutagenesis of C-terminal residues α-Tyr44 to Phe and α-Tyr44 and α-Tyr45 to Phe and Leu produced B800–838 and then B800–826 complexes, respectively;^43^ Raman spectroscopy verified the disruption of this bonding arrangement.^42^ The progressively larger overlap between B800 and the blue-shifted LH2 absorption bands increased the rate of excitation transfer between the two rings of pigment.^68^ When the same mutants were examined in an intact photosystem where LH2 donates energy to the RC–LH1–PufX complex, energy transfer from B800–850, B800–839, and B800–826 LH2 complexes to LH1 slowed from 4.6 ps to 6.2 and 7.0 ps, respectively, due to a decreased spectral overlap between the LH2 donor and the 875 nm absorbing LH1 acceptor.^47^ Slowed rates of forward LH2 to LH1 energy transfer for blue-shifted mutants were accompanied by slower back-transfer of energy from the RC–LH1–PufX core complex to the blue-shifted LH2 antenna,^69^ an observation also made for low-light *Rbl. acidophilus* cells containing B800–820 and core complexes.^70^ It appears that the assembly of a B800–820 antenna, achieved by swapping hydrogen-bonding residues for those that cannot participate in bonding, confers an advantage in low-light conditions by minimizing back-transfer, thereby “concentrating” excitations within cores where they can be trapped at the RC.

## Conclusions

The *Rba. sphaeroides* LH2 complex has featured in numerous spectroscopic, developmental, physiological, and computational studies, aimed at understanding its absorption and energy-transfer functions, the way in which LH2 arrays improve the collection of solar energy under low light and how LH2 arrays influence the architecture, organization, and shape of photosynthetic membranes. Invaluable guides to the structure of this nonameric LH2 complex were provided over 25 years ago by the crystallographic structure of the LH2 from *Rbl. acidophilus*(10,11) and by the 6 Å projection structure of the *Rba. sphaeroides* LH2 complex.^15^ Single-particle cryo-EM has eventually supplied the LH2 structure at a 2.1 Å resolution, high enough to reveal the details of the internal molecular arrangement of carotenoids, BChls, and bonding interactions that establish internal energy-transfer pathways and which enable LH2 to form an adaptable network for gathering excitation energy and transferring it to RC–LH1–PufX complexes.

## Supporting Information Available

Cryo-EM images of the LH2 complex from *Rba. sphaeroides* and resolution calculation of 3D map; qualitative determination of metal ions in the LH2 complexes from *Rbl. acidophilus* and *Rba. sphaeroides* by energy-dispersive X-ray analysis (EDX); absorbance spectra of the LH2 complexes of *Rba. sphaeroides**;* numbering of carbons in spheroidene; and cryo-EM data acquisition, model refinement, and validation statistics (PDF)

## Accession Codes

The cryo-EM density map has been deposited in the World Wide Protein Data Bank (wwPDB) under accession code EMD-13307, and the coordinates have been deposited in the Protein Data Bank (PDB) under accession number 7PBW.
