# Supplementary material for: Cryo-EM Structure of the Rhodobacter sphaeroides Light-Harvesting 2 Complex at 2.1 Å
Source: Biochemistry. 2021 Oct 26;60(44):3302–14. doi: 10.1021/acs.biochem.1c00576 (PMC8775250; doi:10.1021/acs.biochem.1c00576)
Supplement: Supplementary file 1 — bi1c00576_si_001.pdf [file bi1c00576_si_001.pdf]

Supporting Information

The cryo-EM structure of the *Rhodobacter sphaeroides* light-harvesting 2 complex at 2.1 Å

Pu Qian,[‡,†] David J.K. Swainsbury,[†] Tristan I. Croll,[∞] Pablo Castro-Hartmann,[‡] Giorgio Divitini,[#] Kasim Sader,[‡] and C. Neil Hunter[†*]

[‡]Materials and Structural Analysis, Thermo Fisher Scientific, Achtseweg Noord 5, 5651 GG Eindhoven, Netherlands

[∞]Cambridge Institute for Medical Research, University of Cambridge, Cambridge CB2 0XY, UK

[#]Department of Materials Science and Metallurgy, University of Cambridge, Cambridge CB3 0FS, UK

[†]Department of Molecular Biology and Biotechnology, University of Sheffield, Sheffield, S10 2TN, UK

*Email: c.n.hunter@sheffield.ac.uk

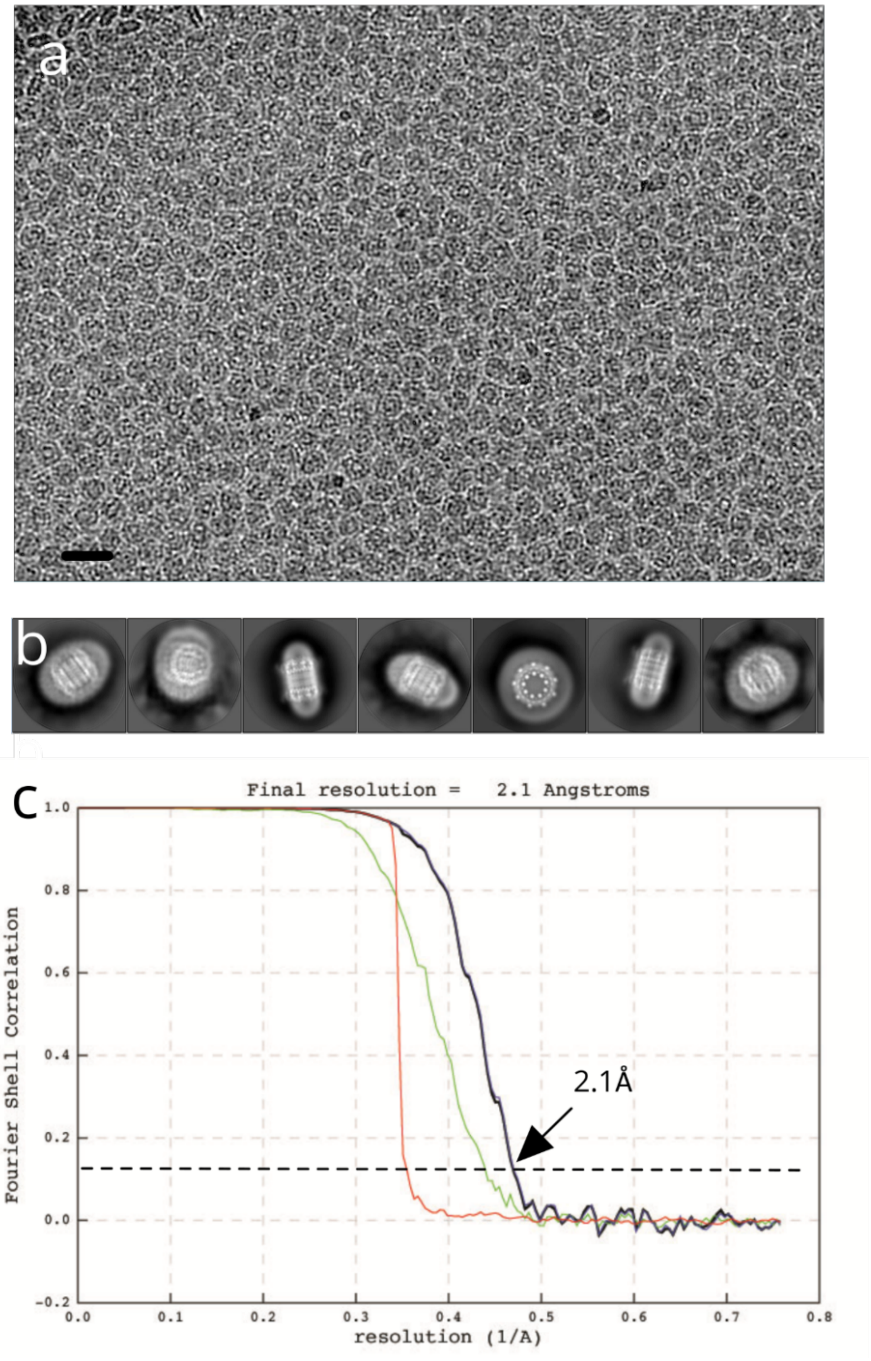

Fig. S1. Cryo-EM images of the LH2 complex from *Rba. sphaeroides* and resolution calculation of 3D map. a) A raw cryo-EM image of the LH2 molecules embedded in vitrified water. The image was 20 Å lowpass filtered with a sigma contrast of 3.0. Scale bar 10 nm. b) Selected 2D classes, showing different orientation of the LH2 molecules in the vitrified ice layer. The box size is 17.8 nm. c) Fourier shell correlation (FSC) curves of the CTF corrected (*black line*), masked (*blue solid line*), unmasked (*green line*), and phase randomized (*red line*) half-maps with C9 symmetry. The 0.143 cutoff is indicated by a dashed line.

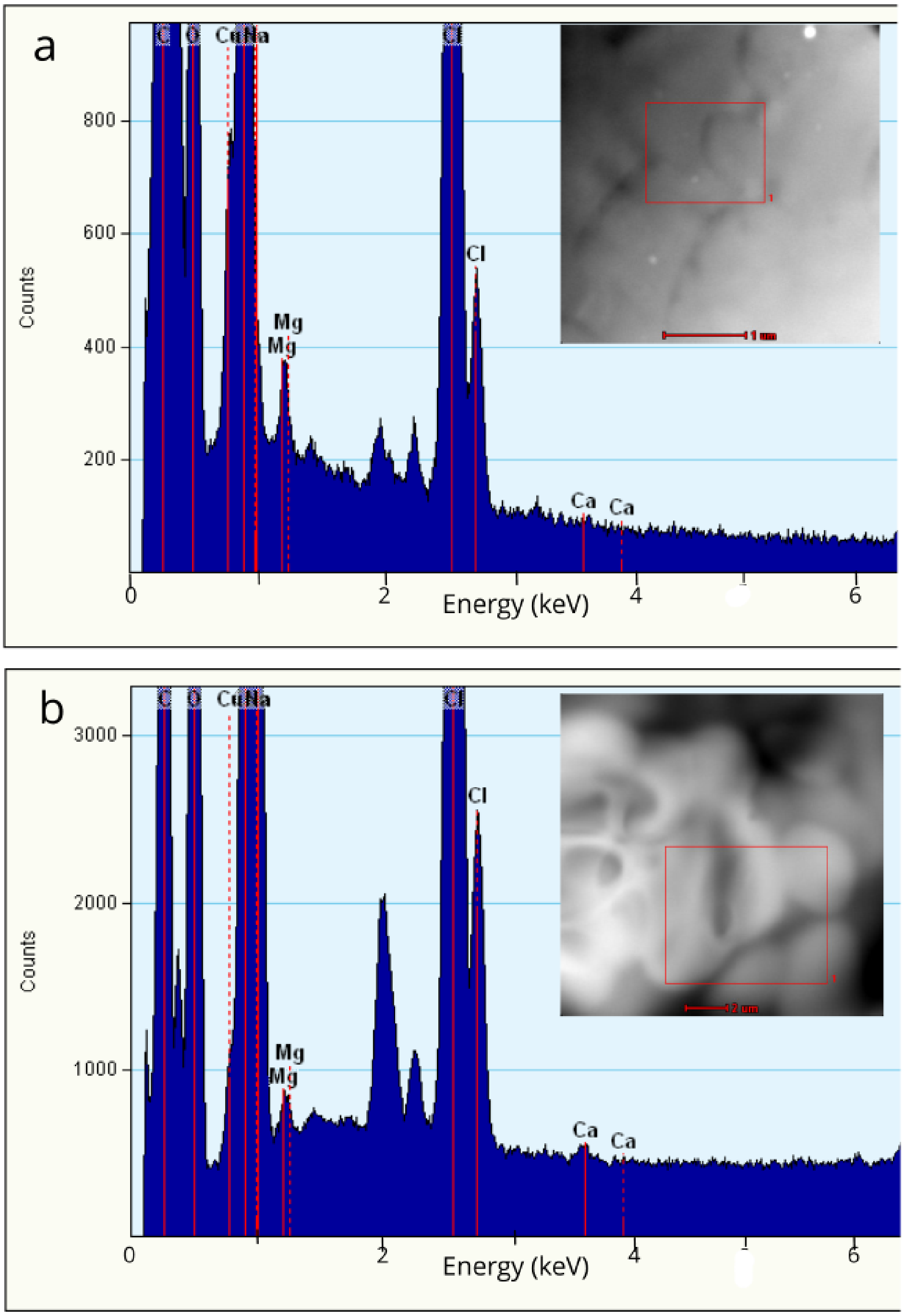

Fig. S2. Qualitative determination of metal ions in the LH2 complexes from *Rbl. acidophilus* and *Rba. sphaeroides* by energy dispersive X-ray analysis (EDX*)*. a, An EDX spectrum from the LH2 of *Rbl. acidophilus***.** Peak positions of magnesium and calcium are indicated. The insert shows a scanned area indicated by a red square with a scale bar. b, EDX spectrum from the LH2 of *Rba. sphaeroides.*

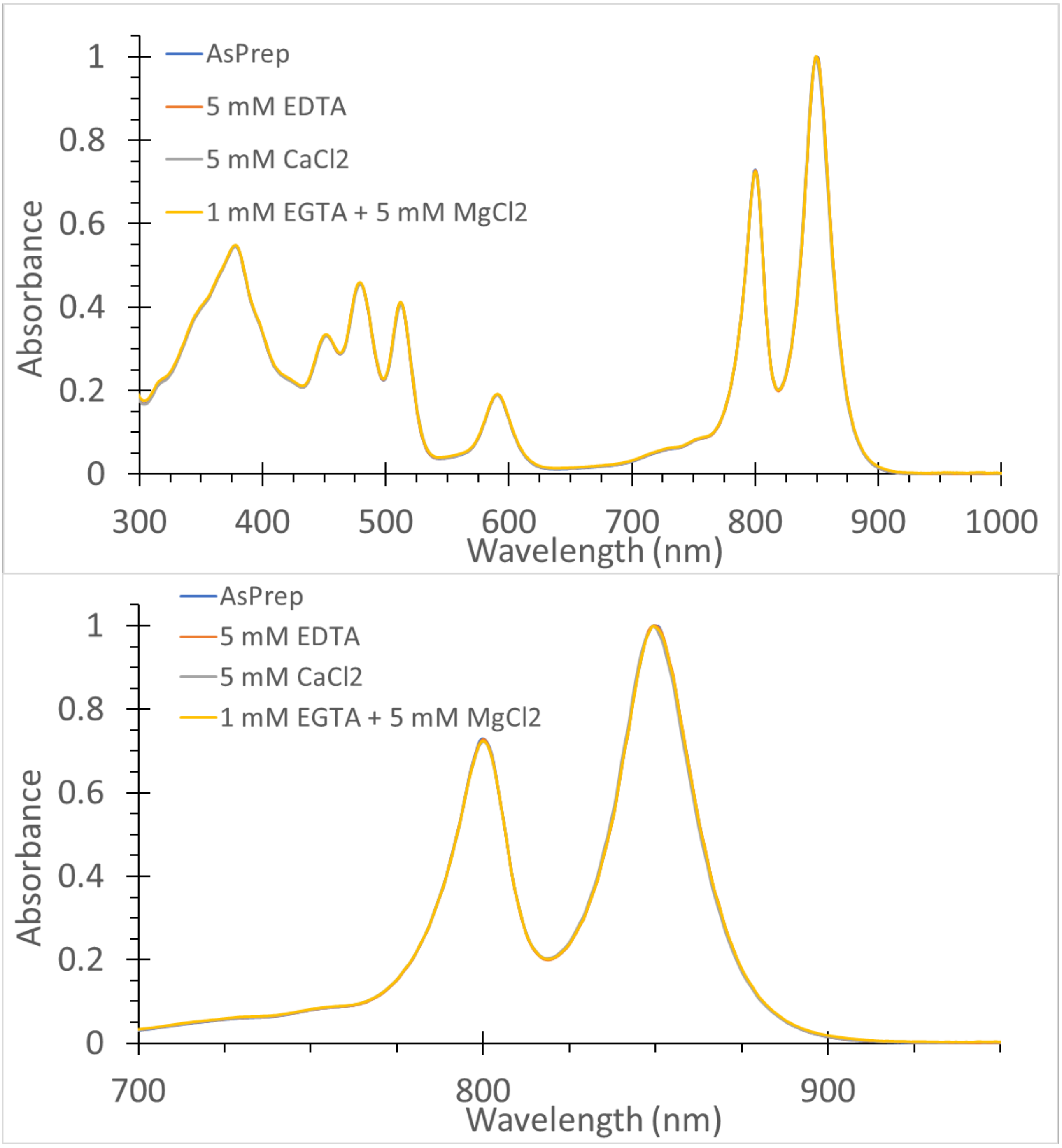

Fig. S3. Absorbance spectra of the LH2 complexes of *Rba. sphaeroides,* recorded from 300 to 1000 nm, with the 700-950 nm region expanded in the lower set of spectra. The spectra of the complexes were unaffected by treatments with EDTA, $CaCl_2$ or EGTA and $MgCl_2$, and so were superimposable.

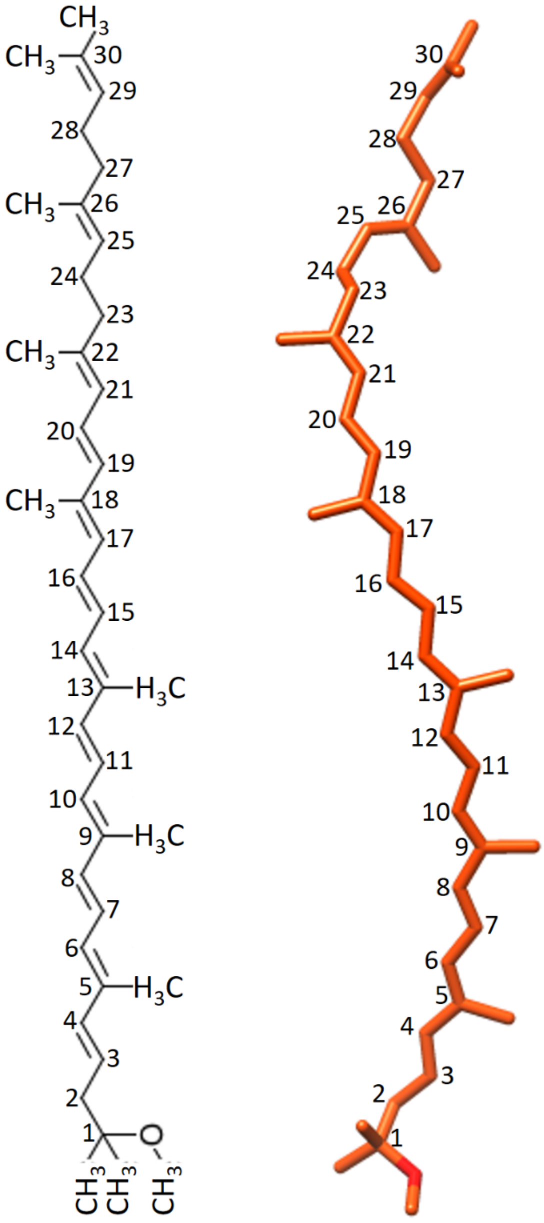

Fig. S4. Numbering of carbons in spheroidene, shown in diagram form (left) and in the all-*trans* conformation (right) adopted within the LH2 complex.

Table S1: Cryo-EM data acquisition, model refinement and validation statistics.

**Protein source**	Photosynthetic bacterium
**Data collection and processing**	
Microscope	ThermoFisher Titan Krios G2
Voltage (kV)	300
Camera	Gatan BioQuantum K3
Energy filter	Yes
Energy filter slit width	20 eV
Magnification	130,000
Defocus range (µm)	-0.8 to -2.2
Mean defocus (µm)	-1.8
Pixel size (Å)	0.66
Electron flux (e^-/ $Å^2$/s)	1.37
Electron fluence (e^-/ $Å^2$)	41.6
Exposure time (sec/frame)	0.034
Electron fluence per frame (e/ $Å^2$/frame)	1.04
Number of frames per movie	40
Number of movies used	3138
Initial no. particle images	1,719,688
Final no. particle images	519,005
Symmetry imposed	C9
Local resolution range	2.0 to 2.5
Resolution of unmasked reconstruction (Å, FSC=0.143)	2.3
Resolution of masked reconstruction (Å, FSC=0.143)	2.1
Specimen temperature	~80K
Particle box size	$(512\ px)^2$
**Refinement and validation**	
Refinement package	COOT, ISOLDE, PHENIX
Initial model	PDB 1NKZ
Model resolution (Å, FSC=0.5)	2.1
Map sharpening B factor ($Å^2$)	-77.06
**Model composition**	
Non-hydrogen atoms	9500
Protein residues	873
Molecular weight (kD)	134.7
Protein B factor ($Å^2$)	11.2
**RMS deviations**	
Bond length (Å)	0.004
Bond angle (^{0})	0.941
**Validation**	
MolProbity score	1.11
Clashscore	3.20
Rotamer outliers (%)	0.00
EMRinger score	7.90
Cb deviations (%)	0.00
CaBLAM outliers (%)	0.00
**Ramachandran plot**	
Favoured (%)	100.00
Allowed (%)	0.00
Disallowed (%)	0.00
Ramachandran Z-score	3.14
**PDB ID**	7PBW
**EMDB ID**	13307
